# Transgenesis and targeted mutagenesis in the human-parasitic nematode *Strongyloides stercoralis*

**DOI:** 10.3389/fpara.2026.1830920

**Published:** 2026-05-07

**Authors:** Matthew S. Moser, Elissa A. Hallem

**Affiliations:** 1 Department of Microbiology, Immunology, and Molecular Genetics, University of California, Los Angeles, Los Angeles, CA, United States; 2 Molecular Biology Interdepartmental PhD Program, University of California, Los Angeles, Los Angeles, CA, United States; 3 Molecular Biology Institute, University of California, Los Angeles, Los Angeles, CA, United States

**Keywords:** CRISPR, human-parasitic helminth, molecular genetics, piggyBac, *Strongyloides stercoralis*, targeted mutagenesis, transgenesis

## Abstract

*Strongyloides stercoralis* is a soil-transmitted helminth that is responsible for an estimated 300–600 million human infections worldwide, mostly in impoverished areas of tropical and sub-tropical countries. To date, *S. stercoralis* is also the only human-parasitic nematode that is amenable to the generation of stable transgenic or mutant lines that can be maintained indefinitely by passage through a laboratory host, the Mongolian gerbil. Because of its health significance and ease of genetic manipulation, *S. stercoralis* has rapidly emerged as a model system for understanding the molecular and cellular basis of nematode parasitism. In this mini-review, we discuss the approaches used to generate transgenic and mutant *S. stercoralis*, with an emphasis on approaches for generating stable transgenic or knockout lines. We also discuss how these approaches are enabling new insights into the basic biology of these highly pathogenic, and sometimes deadly, human parasites.

## Introduction

Soil-transmitted helminths (STHs) are a classification of parasitic nematodes characterized by transmission via ingestion of infective eggs or larvae, or by contact with infective larvae in soil contaminated with feces (Buonfrate et al., 2023; Czeresnia and Weiss, 2022; Loukas et al., 2021). STHs include hookworms in the genera *Necator* and *Ancylostoma*, the giant intestinal roundworm *Ascaris lumbricoides*, the whipworm *Trichuris trichiura*, and the human threadworm *Strongyloides stercoralis* (Buonfrate et al., 2023; Czeresnia and Weiss, 2022; Loukas et al., 2021). These gastrointestinal helminths infect over 1.5 billion people worldwide, causing chronic debilitating conditions, and *S. stercoralis* infections can be fatal in immunocompromised individuals (Buonfrate et al., 2020; Chen et al., 2024; Fleitas et al., 2022; Loukas et al., 2021; Riaz et al., 2020). In addition, children infected with STHs may have stunted growth or nutrient deficiencies, resulting in life-long medical complications (Fauziah et al., 2022; Forrer et al., 2017). Economically disadvantaged individuals living in tropical or subtropical regions with inadequate access to proper sewage and health infrastructure are disproportionately impacted by STHs (Beknazarova et al., 2016; Loukas et al., 2021; Lynn et al., 2021; Magalhães et al., 2023; McKenna et al., 2017). Individuals that come in contact with STH-contaminated soil are infected either via the fecal-oral route (*A. lumbricoides* and *T. trichiura*) or skin penetration (hookworms and *S. stercoralis*) (Buonfrate et al., 2023; Loukas et al., 2021). Some hookworms, and possibly *S. stercoralis*, can also infect when infective larvae are ingested (Buonfrate et al., 2023; Loukas et al., 2021). While ivermectin is administered as a gold-standard anthelmintic treatment, there are currently no prophylactics or vaccines available against any of the STHs, and subpar drug efficacy and resistance are major concerns (Buonfrate et al., 2023; Coffeng et al., 2024; Fissiha and Kinde, 2021; Ng’etich et al., 2023; Sengthong et al., 2021).

*S. stercoralis* is the causative agent of the neglected tropical disease strongyloidiasis, which infects an estimated 300–600 million people globally (Buonfrate et al., 2020; Fleitas et al., 2022). Individuals infected with *S. stercoralis* may experience gastrointestinal distress. However, severe cases arise when individuals experience hyperinfection (high worm burden) or larval dissemination throughout the body, typically as a result of immunosuppression (Buonfrate et al., 2023). Efforts to control infections are complicated in cases of autoinfection, where the parasites chronically reinfect the same host. Treatment for autoinfection requires repeated administration of anthelminthics over week-long intervals; coupled with lack of reliable rapid detection methods, this makes strongyloidiasis a particularly challenging disease to eradicate (Buonfrate et al., 2023; Lo et al., 2025; Luvira et al., 2022; Tamarozzi et al., 2023). Efforts to eliminate skin-penetrating nematodes include improving local sanitation infrastructure, wearing closed-toed shoes, and extensive surveillance programs. However, these methods alone are not sufficient to reduce chronic or disseminated infections (Buonfrate et al., 2023; Lo et al., 2025). Thus, understanding the molecular mechanisms that drive parasite pathogenesis is critical for optimizing the development of targeted prophylactics, diagnostics, and therapeutics.

**Figure 1:**
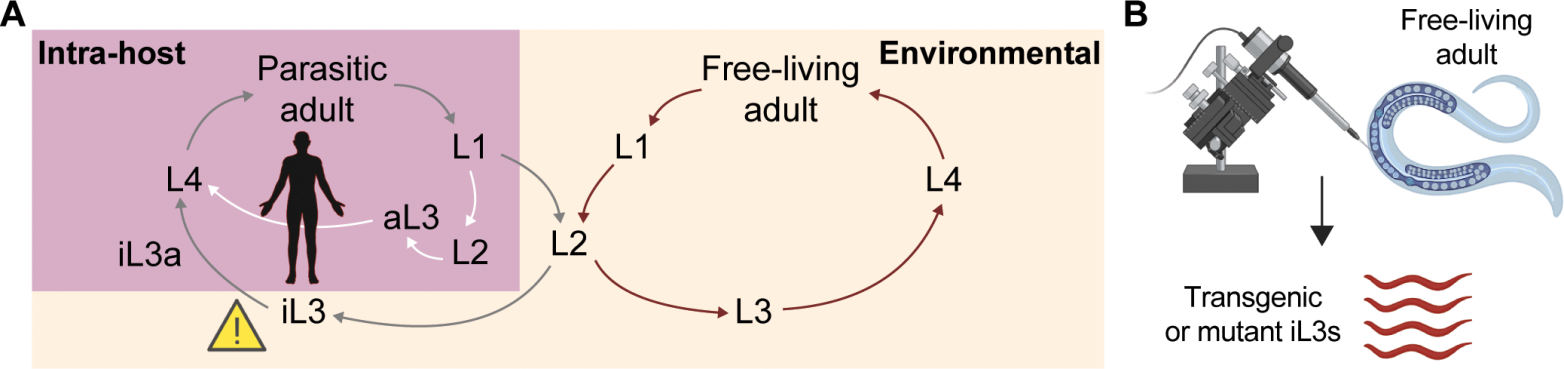
The unique life cycle of *S. stercoralis* allows for the introduction of exogenous DNA into free-living adult females. **(A)** *S. stercoralis* has both intra-host and environmental life stages. Developmentally arrested, soil-dwelling infective third-stage larvae (iL3) are the only infective stage (indicated by the yellow hazard symbol). The iL3s penetrate through skin, migrate through dermal tissue, and resume development (“activate”) to become activated iL3s (iL3as) (Buonfrate et al., 2023). Inside the host, the worms circulate through the bloodstream and respiratory system, ending up as parthenogenic parasitic adult females in the small intestine. The parasitic adults lay eggs that hatch into L1 larvae, some of which are excreted into the environment via feces. These F_1_ larvae, which are termed post-parasitic L1s, can either directly develop into iL3s (left; gray arrows), or they can develop into free-living adult males and females (right; brown arrows). The free-living adults undergo sexual reproduction in the environment, and all of their offspring develop into iL3s (right). In addition, some of the progeny of the parasitic adults do not exit the host and instead develop into autoinfective larvae (aL3s), which complete their life cycle within the same host in a process called autoinfection (white arrows) (Buonfrate et al., 2023). L1-L4 = 1^st^ through 4^th^ larval stages. **(B)** The syncytial gonad of *S. stercoralis* free-living adults (dark blue) can be microinjected with exogenous DNA to generate transgenic or mutant iL3 progeny, making *S. stercoralis* easily amenable to molecular genetic manipulation (Castelletto and Hallem, 2021; Junio et al., 2008; Li et al., 2006; Shao et al., 2017).

*S. stercoralis* has a complex life cycle that consists of both free-living and parasitic stages (**Figure 1A**). Developmentally arrested soil-dwelling infective third-stage larvae (iL3s) locate human hosts using host-emitted sensory cues, including body heat and odorants (Bryant et al., 2018, 2022; Castelletto et al., 2014; Ekobol et al., 2025; Gang et al., 2020; Lopez et al., 2000; Safer et al., 2007). The parasitic life cycle begins when the iL3s release a protease-rich excretory-secretory product and burrow through host skin (Czeresnia and Weiss, 2022; Dishnica et al., 2023; Gomez Gallego et al., 2005; Hunt et al., 2016; McKerrow et al., 1990; Moser and Hallem, 2024). The iL3s then resume development, a process called activation, and migrate through the host circulatory and respiratory systems until ultimately colonizing the crypts of the small intestine as parthenogenetic parasitic adult females (Buonfrate et al., 2023). The progeny of the parasitic adult females can either: 1) develop into larvae that reinfect the same host in a process called autoinfection; 2) exit the host in feces as L1 larvae and develop directly into iL3s; and/or 3) exit the host and undergo a single free-living generation consisting of sexually reproductive adult males and females, whose progeny all develop into iL3s (Buonfrate et al., 2023) (**Figure 1A**).

*S. stercoralis* free-living adults are amenable to transgenesis and targeted mutagenesis using techniques for intragonadal microinjection of exogenous DNA adapted from those used in the free-living model nematode *Caenorhabditis elegans* (**Figure 1B**) (Castelletto et al., 2020; Mendez et al., 2022). These methods are enabled by the sequencing and annotation of the *S. stercoralis* genome, as well as the generation of high-quality RNA-sequencing datasets (Howe et al., 2017; Hunt et al., 2018, 2016, 2017). Here, we review the development of techniques for transgenesis and targeted mutagenesis in *S. stercoralis*. We also discuss how recent technical advances have enabled the generation of the first *S. stercoralis* transgenic and knockout stable lines (Banerjee et al., 2025a; Patel et al., 2025, 2024), thereby greatly facilitating comprehensive studies of gene function in these parasites.

## *C. elegans* as a comparative model for *S. stercoralis*

As a well-established genetic model system, the free-living nematode *C. elegans* offers a powerful starting point for understanding the biology of *S. stercoralis* and other parasitic nematodes. For example, in *C. elegans*, insulin signaling via the insulin receptor Dauer Formation-2 (DAF-2) regulates the entry into and exit from the dauer larval stage, a developmentally arrested third-larval stage that is homologous to the iL3 stage of parasitic nematodes (Hu, 2007; Viney et al., 2005). *S. stercoralis* has a *daf-2* homolog, and aptazyme-mediated disruption of *Sst*-DAF-2 impairs resumption of development by iL3s upon exposure to host-like conditions (Zhang et al., 2025). Thus, insulin signaling regulates larval development in both *C. elegans* and *S. stercoralis*. Similarly, carbon dioxide (CO_2_) detection in both *C. elegans* and *S. stercoralis* requires the receptor guanylate cyclase GCY-9, a putative receptor for CO_2_, and the BAG sensory neurons (named for their bag-like dendritic endings) (Banerjee et al., 2025a; Hallem et al., 2011; Smith et al., 2013). However, it is important to note that *C. elegans* and *S. stercoralis* are distantly related species from different nematode clades, and there is often not a one-to-one correspondence between genes in *C. elegans* and *S. stercoralis* (Ahmed et al., 2022; Blaxter et al., 2016, 1998). For example, the astacin family of zinc metalloproteases is present in both *C. elegans* and *S. stercoralis*; however, while *C. elegans* has 40 astacins, *S. stercoralis* has over 200 (Hunt et al., 2016, 2017; Möhrlen et al., 2003; Moser and Hallem, 2024; Park et al., 2010). The expansion of the astacin family in *S. stercoralis* suggests an important role for these proteins in nematode parasitism (Hunt et al., 2016, 2017; Moser and Hallem, 2024) and highlights the limitations of relying entirely on *C. elegans* to inform studies of parasitic nematodes. The direct study of parasitic nematodes is critical for uncovering the specific molecular and cellular adaptations that enable them to successfully parasitize human hosts.

Fortunately, many of the molecular genetic tools used to engineer transgenic and mutant nematodes originally developed in *C. elegans* have proven to be adaptable to *S. stercoralis*. In *C. elegans*, transgenic and knockout lines can be generated by microinjection of exogenous DNA into the syncytial gonad of young adult hermaphrodites, where it is then taken up into developing oocytes (Evans, 2006; Kim et al., 2022). In the case of transgenesis, both linear and plasmid DNA can be incorporated into extrachromosomal arrays in the F_1_ progeny, which are then inherited transgenerationally (Evans, 2006). In the case of CRISPR-mediated genome editing, either DNA plasmids encoding the single guide RNA and Cas9 endonuclease, or a ribonucleoprotein complex consisting of the Cas9 protein and single guide RNA, are introduced into adult hermaphrodites to generate heritable mutations in the F_1_ progeny (Kim et al., 2022). In the absence of a repair template, Cas9-mediated double-stranded breaks are repaired by non-homologous end joining, typically resulting in small indels that disrupt gene function. However, if DNA containing a repair template is included in the microinjection mix, then the double-stranded break can be precisely repaired by homology-directed repair, resulting in incorporation of the DNA sequence contained in the repair template (Kim et al., 2022). Uses of CRISPR-mediated genome editing include disrupting or editing genes, endogenously tagging genes, and inserting transgenes through targeted integration into the genome (Dickinson and Goldstein, 2016; El Mouridi et al., 2022; Kim et al., 2022).

## Transgenesis in *S. stercoralis*

**Figure 2:**
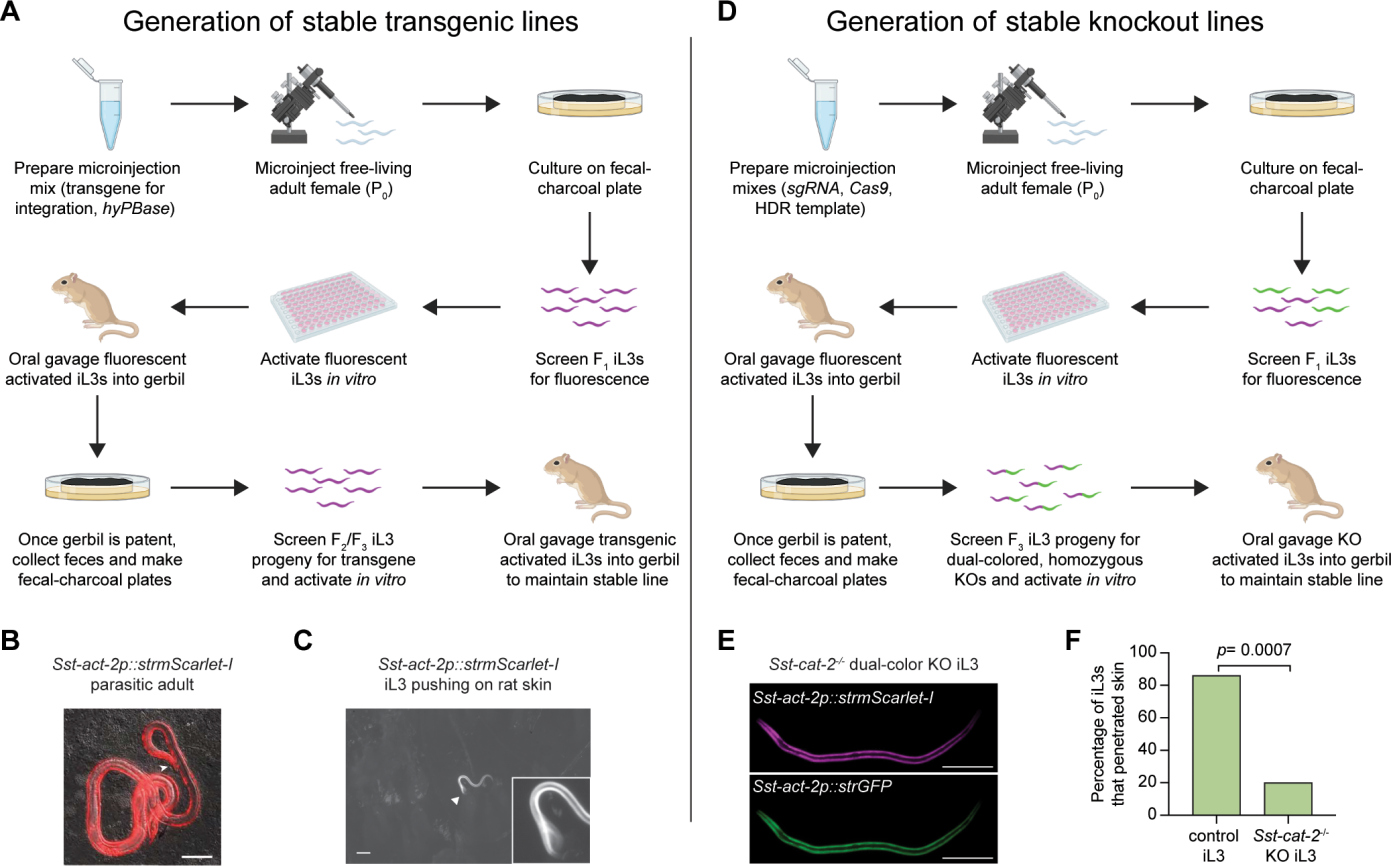
Approach for generating transgenic and knockout stable lines of *S. stercoralis*. A similar approach can be used to generate stable transgenic and knockout lines, with the primary difference being the plasmid mixture that is microinjected into the free-living adult gonad. **(A)** To generate a stable transgenic line, the microinjection mixture contains the following: a vector containing the transgene for integration that is flanked by inverted terminal repeats (ITRs), and a hyperactive *piggyBac* transposase (*hyPBase*) expression vector. Co-injection of the two plasmid vectors will enable integration of the transgene into the genome by hyPBase in a semi-random manner at 5′-TTAA-3′ recognition sites (Patel et al., 2024; Sandoval-Villegas et al., 2021; Yusa, 2015; Yusa et al., 2011). Transgenic F_1_ progeny are collected from fecal-charcoal plates and screened for expression of the transgene before undergoing *in vitro* activation. Activated iL3s are then inoculated into a gerbil via oral gavage. Once the gerbil is patent, feces from the gerbil are collected and mixed into a fecal-charcoal plate. F_2_/F_3_ iL3s are then isolated, screened for expression of the transgene, and activated *in vitro*. These activated iL3s are gavaged into a new gerbil to establish and maintain a stable line of transgenic *S. stercoralis* (Patel et al., 2024). **(B)** A transgenic *S. stercoralis* parasitic adult expressing an *Sst-act-2p::strmScarlet-I* transgene, which drives expression of mScarlet-I in body-wall muscle. Scale bar = 100 µm. Figure is from Patel et al., 2024 (Patel et al., 2024). **(C)** The *Sst-act-2p::strmScarlet-I* transgenic stable line has been used to visualize iL3 behavior on skin. Image shows an *Sst-act-2p::strmScarlet-I* iL3 on rat skin, where the head of the iL3 is pushing against the skin surface. Arrowhead indicates the head of the worm. The head of the worm is enlarged in the inset and is out of focus because the iL3 is moving its head to actively push against the skin. Scale bar = 100 µm; inset is magnified 4x. Figure is from Patel et al., 2025 (Patel et al., 2025). **(D)** To generate a stable knockout line, the microinjection mixture contains the following: a plasmid encoding a single guide RNA for an identified CRISPR site; the Cas9 endonuclease expression vector; and one of two homology-directed repair constructs (HDRs) containing either an *Sst-act-2p::strmScarlet-I* or *Sst-act-2p::strGFP* reporter, which drives red or green fluorescence in body-wall muscle (alternatively, one of these reporters can be used in combination with a reporter that drives blue fluorescence) (Banerjee et al., 2025a, 2025b; Patel et al., 2025). The plasmid mixes containing either the red HDR construct or the green HDR construct are injected in tandem into separate groups of free-living females. The process for generating a stable knockout line is then similar to that for generating a stable transgenic line, except that F_1_ iL3s with either red or green fluorescence in their body-wall muscle are collected, pooled, activated, and then oral-gavaged into the same gerbil. Free-living red or green F_2_ progeny from that gerbil will mate on the fecal-charcoal plate to generate F_3_ iL3s that are both red and green; these F_3_ iL3s are homozygous knockouts, with the red fluorophore integrated into one allele of the target gene and the green fluorophore integrated into the other allele. **(E)** An *S. stercoralis* iL3 containing a homozygous disruption of the tyrosine hydroxylase gene *Sst-cat-2*, which is involved in dopamine biosynthesis. The iL3 has an *Sst-act-2p::strmScarlet-I* transgene integrated into one allele of the *Sst-cat-2* gene and an *Sst-act-2p::strGFP* transgene integrated into the other allele, so both red and green fluorescence are observed throughout the body-wall muscle. Scale bar = 100 µm. Image is from Patel et al., 2025 (Patel et al., 2025). **(F)** An example of how knockout iL3s can be used to gain insight into the molecular basis of behavior. Graph shows the results of a skin-penetration behavioral assay, in which iL3s are placed on human skin and their behavior is video-recorded and then analyzed *post hoc*. The *Sst-cat-2^-/-^* iL3s are defective in their ability to penetrate human skin: whereas ~80% of the control iL3s penetrate human skin within 10 min, only ~20% of the *Sst-cat-2^-/-^* iL3s penetrate. These results demonstrate a critical role for dopamine signaling in driving skin-penetration behavior. n = 15 iL3s per genotype. *p* = 0.0007, two-tailed Fisher’s exact test. Graph is from Patel et al., 2025 (Patel et al., 2025). For **B, C**, and **E**, “*str*” indicates that the fluorophore gene is codon-optimized for expression in *Strongyloides* species (Banerjee et al., 2025a, 2025b; Patel et al., 2025). Schematics in **A** and **D** are adapted from Patel et al., 2024 and Banerjee et al., 2025b (Patel et al., 2024; Banerjee et al., 2025b).

Like *C. elegans* adults, *S. stercoralis* free-living adults have a syncytial gonad; thus, exogenous DNA can be introduced by intragonadal microinjection using techniques adapted from *C. elegans* (**Figure 2A**) (Castelletto and Hallem, 2021; Junio et al., 2008; Li et al., 2006; Shao et al., 2017). The generation of transgenics via intragonadal microinjection is not yet feasible in parasitic nematodes such as hookworms that lack a free-living (and therefore easily accessible) adult life stage. However, heritable transgenesis via intragonadal microinjection has been successfully applied to the rat-parasitic nematode *Strongyloides ratti* and the possum-parasitic nematode *Parastrongyloides trichosuri*, both of which have a life cycle that includes a free-living adult stage similar to that of *S. stercoralis* (Grant et al., 2006; Li et al., 2011; Lok, 2012; Lok et al., 2017; Shao et al., 2012). Successful transgenesis requires the use of endogenous promoters and regulatory elements (Junio et al., 2008; Li et al., 2006), and transgenesis rates are greatly improved by the codon optimization of transgenes (Bryant and Hallem, 2021; Patel et al., 2024). It is important to note that manual curation of *S. stercoralis* genes is also often critical to ensure appropriate transgene design (Bryant et al., 2024).

While *C. elegans* can express transgenes from extrachromosomal arrays across generations (Evans, 2006), this feature is not shared with *S. stercoralis*. In *Strongyloides*, extrachromosomal arrays are silenced after the F_1_ generation (Junio et al., 2008; Lok, 2013; Shao et al., 2012). Thus, heritable expression in *Strongyloides* requires genomic integration of transgenes. This has been achieved using the *piggyBac* system (Lok, 2013, 2012; Shao et al., 2012, 2017). The piggyBac transposase, originally derived from the cabbage looper moth *Trichoplusia ni* (Yusa, 2015), drives chromosomal integration by excising DNA sequences flanked by inverted terminal repeats (ITRs) and integrating them into the genome at sites containing TTAA motifs (Sandoval-Villegas et al., 2021). To integrate transgenes using the *piggyBac* system, two plasmids are microinjected: one encodes the transgene of interest flanked by ITRs and the other encodes the piggyBac transposase. Advantages of the *piggyBac* system include the ability to integrate large transposons of over 100 kb and the high copy number of integrated transgenes, resulting in robust transgene expression (Sandoval-Villegas et al., 2021). In *S. ratti*, copy number estimates ranged from ~30–50 per genome (Shao et al., 2012).

Until recently, even with the use of *piggyBac*, the generation of stable transgenic lines that could be maintained indefinitely across generations was feasible with *S. ratti* but not *S. stercoralis* (Lok, 2012). This is because maintenance of *S. stercoralis* requires passaging infective larvae through a laboratory host such as the Mongolian gerbil (*Meriones unguiculatus*), and subcutaneous injection of 1,000-2,000 iL3s per gerbil was required to establish a patent infection (Nolan et al., 1993). The relatively low transgenesis rate of *S. stercoralis* (typically, transgenic larvae comprise <5% of the F_1_ iL3 population) made generating ~1,000 transgenic F_1_ iL3s impractical (Castelletto and Hallem, 2021). In one study, sufficient transgenic *S. stercoralis* F_1_ iL3s were collected and used to successfully infect a gerbil; however, only two transgenic larvae were obtained in the F_2_ generation, precluding further host passage (Lok, 2012). Thus, studies of transgenic *S. stercoralis* were limited to small numbers of transgenic F_1_ iL3s.

These limitations were recently overcome to generate the first stable transgenic lines of *S. stercoralis* (**Figure 2A**) (Patel et al., 2024). Stable transgenesis was achieved through several modifications to the original transgenesis protocol: 1) A hyperactive piggyBac transposase, hyPBase, was used for genomic integration, since hyPBase was found to have a ~9-fold higher integration rate in mammalian cells (Yusa et al., 2011); 2) the *hyPBase* transgene was codon-optimized for expression in *S. stercoralis*; 3) transgenic iL3s were subjected to *in vitro* activation, a process whereby the iL3s are exposed to host-like conditions that cause them to initiate development outside the host (Gang et al., 2020; Stoltzfus et al., 2014, 2012); and 4) the iL3s were introduced into the gerbil by oral gavage instead of subcutaneous injection (Patel et al., 2024). Transgenic worms were then isolated from gerbil feces (Patel et al., 2024). Infecting gerbils with activated iL3s by oral gavage reduced the number of larvae required to establish an infection from ~1,000 to ~100, making it more easily feasible to obtain sufficient numbers of transgenic larvae by intragonadal microinjection (Patel et al., 2024). Using this approach, transgenic *S. stercoralis*, with stable transgenic phenotypes, can be propagated in gerbils across multiple generations. The generation of stable transgenic lines offers numerous experimental advantages, including the ability to propagate transgenic worms through gerbil hosts indefinitely, less mosaic expression patterns, and the ability to generate large numbers of transgenic worms of any life stage (**Figure 2B**) (Patel et al., 2024).

Transgenic *S. stercoralis* have been used extensively in many different studies. *S. stercoralis* iL3s expressing transcriptional reporter constructs, in which an endogenous promoter sequence is used to drive expression of a fluorophore gene, have been used to examine gene expression patterns in multiple contexts (Banerjee et al., 2025a; Bryant et al., 2018, 2022; Castelletto et al., 2009; Cheong et al., 2021; Lok, 2012; Patel et al., 2025, 2024; Stoltzfus et al., 2012; Zhou et al., 2023). Transgenic *S. stercoralis* have also been used to study gene function through expression of dominant negative alleles (Castelletto et al., 2009) and, more recently, through the use of aptazymes to generate conditional gene knockdowns (Zhang et al., 2025). Transgenesis has also been used to genetically target specific subsets of neurons for studies of neuronal function, including neuronal silencing coupled with quantitative behavioral analysis (Banerjee et al., 2025a; Bryant et al., 2022; Patel et al., 2025) and *in vivo* calcium imaging of neuronal activity (Banerjee et al., 2025a; Bryant et al., 2022). Finally, stable transgenic lines that express the fluorophore mScarlet-I in body-wall muscle (**Figure 2B**) have been essential for studies of skin penetration by enabling visualization of the normally translucent worms on the surface of the skin (**Figure 2C**) (Abell et al., 2025; Patel et al., 2025).

Although generating transgenic lines using the *piggyBac* system is straightforward and efficient, there are several disadvantages of this approach. First, integration with *piggyBac* occurs in a semi-random manner that has the potential to cause unintentional gene disruptions (Sandoval-Villegas et al., 2021; Yusa, 2015). Additionally, the number of transgene copies that are integrated into the genome cannot be controlled, making it impossible to mimic endogenous expression levels (Sandoval-Villegas et al., 2021; Yusa, 2015). Finally, because the transgenes integrate at high copy number, it is difficult to make the transgene homozygous at every integration site to obtain a true homozygous line. As a result, maintaining a stable transgenic line requires the periodic selection of bright transgenics to ensure that non-transgenic worms or low-expressing worms do not increase in population frequency over successive host passages. CRISPR/Cas9-mediated genomic integration, described below, offers a powerful alternative for targeted genomic integration in cases where lower copy number is required or less bright transgene expression is acceptable.

## Targeted mutagenesis in *S. stercoralis*

*S. stercoralis* is also amenable to CRISPR/Cas9-mediated targeted mutagenesis using the same intragonadal microinjection technique that is used to make transgenics (Gang et al., 2017; Lok et al., 2017). Typically, plasmids encoding the single guide RNA(s) for targeting the gene of interest, the Cas9 endonuclease, and a template for homology-directed repair (HDR) that encodes a fluorescent reporter are microinjected into the syncytial gonad of free-living females; homozygous iL3s can then be found in the F_1_ generation (Banerjee et al., 2025a; Bryant et al., 2018, 2022; Cheong et al., 2021; Gang et al., 2017, 2020). The Cas9 target sites that result in efficient editing are the same in *S. stercoralis* and *C. elegans* (Banerjee et al., 2025b; Farboud, 2017), making target site selection straightforward. Among the F_1_ progeny from microinjected females, candidate mutants can be identified based on expression of the fluorescent marker, and from this population, homozygous mutants can be identified by single-worm PCR genotyping (Gang et al., 2017). CRISPR-induced mutations can also be obtained by injecting the CRISPR components as a ribonucleoprotein complex (RNP) (Adams et al., 2019; Gang et al., 2017); however, in at least some cases, homology-directed repair appears to be more efficient when plasmids are used instead of RNPs (Gang et al., 2017). Additionally, a template for homology-directed repair does not need to be included; however, in the absence of a repair template, large deletions rather than small indels have been observed (Gang et al., 2017).

In these earlier CRISPR studies, stable mutant lines were not generated. Rather, the F_1_ iL3 progeny of microinjected females were screened for expression of the fluorescent reporter from the HDR construct, subjected to single-worm microscopy or behavioral analysis, and then genotyped *post hoc* to identify homozygous knockouts (Banerjee et al., 2025a; Bryant et al., 2018, 2022; Cheong et al., 2021; Gang et al., 2017, 2020). This approach was time-consuming and labor-intensive because a majority of the candidate mutants tested were not homozygous knockouts – some were heterozygous for the mutation, some were mosaic, and some were wild type but expressed the fluorescent reporter from an extrachromosomal array. Only a few homozygous knockouts could be obtained from a given microinjection experiment, and the CRISPR pipeline had to be repeated multiple times to obtain sufficient sample sizes.

More recently, an approach for developing the first stable knockout lines was developed (**Figure 2D**) (Banerjee et al., 2025a, 2025b; Patel et al., 2025), using a modification of the approach for making stable transgenic lines (Patel et al., 2024). To make stable knockout lines, two separate groups of free-living females are microinjected in parallel; one group receives a microinjection cocktail that includes an HDR construct with a red fluorophore and a second group receives a microinjection cocktail that includes an HDR construct with a green fluorophore (alternatively, a blue fluorophore can be used instead of the red or green fluorophore). The F_1_ iL3 progeny from these two groups of microinjected females are screened for red or green fluorescence, and the red or green iL3s are then pooled. Following the same strategy used for generating stable transgenic lines (Patel et al., 2024), these red or green iL3s are then activated *in vitro*, pooled, and introduced into a gerbil host by oral gavage. Once the gerbil is patent, it will shed F_2_ progeny, some of which will develop into red or green free-living adults. The free-living adults will then mate on feces to generate red/green dual-color F_3_ iL3s. These dual-color iL3s are homozygotes, with a red fluorophore integrated into one allele of the target gene and a green fluorophore integrated into the other allele (**Figure 2E**). Notably, because extrachromosomal arrays are silenced after the F_1_ generation in *S. stercoralis*, all fluorescence in subsequent generations reflects integrated transgenes, and thus all red/green worms are homozygous knockouts. These homozygotes can then be propagated by subsequent passages through a gerbil (**Figure 2D**). Alternatively, in cases where homozygotes show infection defects, the worms can be propagated through gerbil hosts as heterozygotes, and homozygotes can be obtained as needed by mating red free-living females with green free-living males, or vice versa (Banerjee et al., 2025b; Patel et al., 2025). With stable mutant lines, it is possible to obtain large numbers of homozygous mutant worms of any life stage. This approach was recently used to demonstrate a role for the receptor guanylate cyclase gene *Sst-gcy-9* in mediating CO_2_ response (Banerjee et al., 2025a), and to demonstrate a role for the tyrosine hydroxylase gene *Sst-cat-2* in driving skin-penetration behavior (**Figures 2E, F**) (Patel et al., 2025). It should now be possible to use this approach to study nearly any non-lethal gene in *S. stercoralis*.

## Conclusions and future directions

The ability to make stable transgenic and knockout lines of *S. stercoralis* has greatly accelerated mechanistic studies of these parasites. However, several important directions for future technical development remain. First, *S. stercoralis* cannot yet be maintained without an animal host, and host passage is both expensive and time-consuming. An *in vitro* culturing system would be invaluable for the low-cost maintenance of stable knockout and transgenic lines. Second, although most genes can now be targeted for mutagenesis using CRISPR, optimal Cas9 target sites, which include the 5′-GN(17)GG-3′ consensus sequence and a 5′-NGG-3′ protospacer adjacent motif (PAM) (Banerjee et al., 2025b; Farboud, 2017; Farboud and Meyer, 2015), are limited in the AT-rich genome of *S. stercoralis*. Thus, the use of other Cas proteins that favor AT-rich sequences, such as the Cas12 proteins (Yang et al., 2024), would likely enhance genome editing efficiency in *S. stercoralis*. In addition, the implementation of other CRISPR/Cas systems for activating or repressing transcription, editing RNA sequences, or altering RNA levels (Knott and Doudna, 2018) would further expand the *S. stercoralis* molecular genetic toolkit. Additional methods for conditional knockdown of gene function, such as the auxin inducible degradation system (Ashley et al., 2021; Zhang et al., 2015), would also be useful, especially in cases where gene knockout is lethal. Third, bipartite expression systems such as the Cre/Lox (Flavell et al., 2013), Q (Monsalve et al., 2019) and Split cGAL (Wang et al., 2017) systems would be invaluable for precise, inducible genetic targeting of specific cells or subsets of cells. These approaches should be easily adaptable to *S. stercoralis* using current transgenesis or CRISPR editing methods. Fourth, the development of additional transcriptional reporters that can be used as markers for CRISPR mutagenesis would make it straightforward to generate double and triple knockout lines by introducing multiple mutations sequentially. Fifth, the identification of *S. stercoralis* genomic safe harbor sites would enable the specific, targeted integration of transgenes using CRISPR without disruption to neighboring genes or uncontrolled copy number; this approach is routinely used for transgene integration in *C. elegans* (El Mouridi et al., 2022; Malaiwong et al., 2023; Yoshina et al., 2016). Finally, as an increasing number of stable transgenic and knockout lines of *S. stercoralis* are generated, an efficient cryopreservation protocol will be essential, given that maintenance of *S. stercoralis* stable lines in gerbils is expensive and labor-intensive. Cryopreservation protocols are already available for some parasitic nematodes, including hookworms (Li et al., 2023; Vacca et al., 2024), and may be adaptable to *S. stercoralis*. The addition of these methods to the *S. stercoralis* toolkit would greatly facilitate studies of the molecular and cellular mechanisms that enable parasitic nematodes to find, invade, and infect human hosts.

## References

[B1] Abell C. R. Patel R. Hallem E. A. (2025). *Strongyloides* species exhibit distinct behaviors on the skin of different mammals. BMC Infect. Dis. 25, 1233. doi: 10.6019/s-biad2189 41044513 PMC12495621

[B2] Adams S. Pathak P. Shao H. Lok J. B. Pires-daSilva A. (2019). Liposome-based transfection enhances RNAi and CRISPR-mediated mutagenesis in non-model nematode systems. Sci. Rep. 9, 483. doi: 10.1038/s41598-018-37036-1. PMID: 30679624 PMC6345965

[B3] Ahmed M. Roberts N. G. Adediran F. Smythe A. B. Kocot K. M. Holovachov O. (2022). Phylogenomic analysis of the phylum Nematoda: conflicts and congruences with morphology, 18S rRNA, and mitogenomes. Front. Ecol. Evol. 9, 769565. doi: 10.3389/fevo.2021.769565

[B4] Ashley G. E. Duong T. Levenson M. T. Martinez M. A. Q. Johnson L. C. Hibshman J. D. . (2021). An expanded auxin-inducible degron toolkit for *Caenorhabditis elegans*. Genetics 217, iyab006. doi: 10.1093/genetics/iyab006. PMID: 33677541 PMC8045686

[B5] Banerjee N. Gang S. S. Castelletto M. L. Walsh B. Ruiz F. Hallem E. A. (2025a). Carbon dioxide shapes parasite-host interactions in a human-infective nematode. Curr. Biol. 35, 277–286. doi: 10.1016/j.cub.2024.11.036. PMID: 39719698 PMC11753939

[B6] Banerjee N. Walsh B. Patel R. Castelletto M. L. Hallem E. A. (2025b). Protocol to generate stable knockout lines in the human-parasitic nematode *Strongyloides stercoralis*. STAR Protoc. 6, 104201. doi: 10.1016/j.xpro.2025.104201. PMID: 41240344 PMC12663616

[B7] Beknazarova M. Whiley H. Ross K. (2016). Strongyloidiasis: a disease of socioeconomic disadvantage. Int. J. Environ. Res. Public Health 13, 517. doi: 10.3390/ijerph13050517. PMID: 27213420 PMC4881142

[B8] Blaxter M. L. De Ley P. Garey J. R. Liu L. X. Scheldeman P. Vierstraete A. . (1998). A molecular evolutionary framework for the phylum Nematoda. Nature 392, 71–75. doi: 10.1038/32160. PMID: 9510248

[B9] Blaxter M. Koutsovoulos G. Jones M. Kumar S. Elsworth B. (2016). “ Phylogenomics of nematoda,” in Next Generation Systematics. Eds. Olson P. D. Hughes J. Cotton J. A. (Cambridge: Cambridge University Press), 62–82.

[B10] Bryant A. S. Akimori D. Stoltzfus J. D. C. Hallem E. A. (2024). A standard workflow for community-driven manual curation of *Strongyloides* genome annotations. Philos. Trans. R. Soc Lond. B. Biol. Sci. 379, 20220443. doi: 10.1098/rstb.2022.0443. PMID: 38008112 PMC10676816

[B11] Bryant A. S. Hallem E. A. (2021). The Wild Worm Codon Adapter: a web tool for automated codon adaptation of transgenes for expression in non-*Caenorhabditis* nematodes. G3 11, jkab146. doi: 10.1093/g3journal/jkab146. PMID: 33914084 PMC8496300

[B12] Bryant A. S. Ruiz F. Gang S. S. Castelletto M. L. Lopez J. B. Hallem E. A. (2018). A critical role for thermosensation in host seeking by skin-penetrating nematodes. Curr. Biol. 28, 2338–2347. doi: 10.1016/j.cub.2018.05.063. PMID: 30017486 PMC6091634

[B13] Bryant A. S. Ruiz F. Lee J. Hallem E. A. (2022). The neural basis of heat seeking in a human-infective parasitic worm. Curr. Biol. 32, 2206–2221. doi: 10.1016/j.cub.2022.04.010. PMID: 35483361 PMC9158753

[B14] Buonfrate D. Bisanzio D. Giorli G. Odermatt P. Furst T. Greenaway C. . (2020). The global prevalence of *Strongyloides stercoralis* infection. Pathogens 9, 468. doi: 10.3390/pathogens9060468. PMID: 32545787 PMC7349647

[B15] Buonfrate D. Bradbury R. S. Watts M. R. Bisoffi Z. (2023). Human strongyloidiasis: complexities and pathways forward. Clin. Microbiol. Rev. 36, e0003323. doi: 10.1128/cmr.00033-23. PMID: 37937980 PMC10732074

[B16] Castelletto M. L. Gang S. S. Hallem E. A. (2020). Recent advances in functional genomics for parasitic nematodes of mammals. J. Exp. Biol. 223, jeb206482. doi: 10.1242/jeb.206482. PMID: 32034038 PMC7790190

[B17] Castelletto M. L. Gang S. S. Okubo R. P. Tselikova A. A. Nolan T. J. Platzer E. G. . (2014). Diverse host-seeking behaviors of skin-penetrating nematodes. PloS Pathog. 10, e1004305. doi: 10.1371/journal.ppat.1004305. PMID: 25121736 PMC4133384

[B18] Castelletto M. L. Hallem E. A. (2021). Generating transgenics and knockouts in *Strongyloides* species by microinjection. J. Vis. Exp. 176, e63023. doi: 10.3791/63023-v PMC9109651 34694289

[B19] Castelletto M. L. Massey H. C. Lok J. B. (2009). Morphogenesis of *Strongyloides stercoralis* infective larvae requires the DAF-16 ortholog FKTF-1. PloS Pathog. 5, e1000370. doi: 10.1371/journal.ppat.1000370. PMID: 19360119 PMC2660150

[B20] Chen J. Gong Y. Chen Q. Li S. Zhou Y. (2024). Global burden of soil-transmitted helminth infections 1990-2021. Infect. Dis. Poverty 13, 77. doi: 10.1186/s40249-024-01238-9. PMID: 39444032 PMC11515461

[B21] Cheong M. C. Wang Z. Jaleta T. G. Li X. Lok J. B. Kliewer S. A. . (2021). Identification of a nuclear receptor/coactivator developmental signaling pathway in the nematode parasite *Strongyloides stercoralis*. Proc. Natl. Acad. Sci. U.S.A. 118, e2021864118. doi: 10.1073/pnas.2021864118. PMID: 33602820 PMC7923533

[B22] Coffeng L. E. Stolk W. A. de Vlas S. J. (2024). Predicting the risk and speed of drug resistance emerging in soil-transmitted helminths during preventive chemotherapy. Nat. Commun. 15, 1099. doi: 10.1038/s41467-024-45027-2. PMID: 38321011 PMC10847116

[B23] Czeresnia J. M. Weiss L. M. (2022). Strongyloides stercoralis. Lung 200, 141–148. doi: 10.1007/s00408-022-00528-z 35396957 PMC8994069

[B24] Dickinson D. J. Goldstein B. (2016). CRISPR-based methods for *Caenorhabditis elegans* genome engineering. Genetics 202, 885–901. doi: 10.1534/genetics.115.182162. PMID: 26953268 PMC4788126

[B25] Dishnica K. Piubelli C. Manfredi M. Kondaveeti R. T. Longoni S. S. Degani M. . (2023). Novel insights into the somatic proteome of *Strongyloides stercoralis* infective third-stage larvae. Parasit Vectors 16, 45. doi: 10.1186/s13071-023-05675-7. PMID: 36721249 PMC9890704

[B26] Ekobol N. Boonjaraspinyo S. Eamudomkarn C. Boonmars T. (2025). Tricky with heat and salt: soil factors, thermotaxis, and potential for heat-saline agar trapping of *Strongyloides* larvae. Biol. (Basel) 14, 559. doi: 10.3390/biology14050559. PMID: 40427748 PMC12109420

[B27] El Mouridi S. Alkhaldi F. Frokjaer-Jensen C. (2022). Modular safe-harbor transgene insertion for targeted single-copy and extrachromosomal array integration in *Caenorhabditis elegans*. G3 (Bethesda) 12, jkac184. doi: 10.1093/g3journal/jkac184. PMID: 35900171 PMC9434227

[B28] Evans T. C. (2006). “ Transformation and microinjection,” in WormBook. Ed. Ambros V. , (Pasadena: WormBook), 1–15. doi: 10.1895/wormbook.1.108.1

[B29] Farboud B. (2017). Targeted genome editing in *Caenorhabditis elegans* using CRISPR/Cas9. Wiley Interdiscip. Rev. Dev. Biol. 6, e287. doi: 10.1002/wdev.287. PMID: 28810059

[B30] Farboud B. Meyer B. J. (2015). Dramatic enhancement of genome editing by CRISPR/Cas9 through improved guide RNA design. Genetics 199, 959–971. doi: 10.1534/genetics.115.175166. PMID: 25695951 PMC4391549

[B31] Fauziah N. Ar-Rizqi M. A. Hana S. Patahuddin N. M. Diptyanusa A. (2022). Stunting as a risk factor of soil-transmitted helminthiasis in children: a literature review. Interdiscip. Perspect. Infect. Dis. 2022, 8929025. doi: 10.1155/2022/8929025. PMID: 35967932 PMC9365611

[B32] Fissiha W. Kinde M. Z. (2021). Anthelmintic resistance and its mechanism: a review. Infect. Drug Resist. 14, 5403–5410. doi: 10.2147/idr.s332378. PMID: 34938088 PMC8687516

[B33] Flavell S. W. Pokala N. Macosko E. Z. Albrecht D. R. Larsch J. Bargmann C. I. (2013). Serotonin and the neuropeptide PDF initiate and extend opposing behavioral states in *C. elegans*. Cell 154, 1023–1035. doi: 10.1016/j.cell.2013.08.001. PMID: 23972393 PMC3942133

[B34] Fleitas P. E. Kehl S. D. Lopez W. Travacio M. Nieves E. Gil J. F. . (2022). Mapping the global distribution of *Strongyloides stercoralis* and hookworms by ecological niche modeling. Parasit Vectors 15, 197. doi: 10.1186/s13071-022-05284-w. PMID: 35676740 PMC9178904

[B35] Forrer A. Khieu V. Schar F. Hattendorf J. Marti H. Neumayr A. . (2017). *Strongyloides stercoralis* is associated with significant morbidity in rural Cambodia, including stunting in children. PloS Negl.Trop. Dis. 11, e0005685. doi: 10.1371/journal.pntd.0005685 29059195 PMC5695629

[B36] Gang S. S. Castelletto M. L. Bryant A. S. Yang E. Mancuso N. Lopez J. B. . (2017). Targeted mutagenesis in a human-parasitic nematode. PloS Pathog. 13, e1006675. doi: 10.1371/journal.ppat.1006675. PMID: 29016680 PMC5650185

[B37] Gang S. S. Castelletto M. L. Yang E. Ruiz F. Brown T. M. Bryant A. S. . (2020). Chemosensory mechanisms of host seeking and infectivity in skin-penetrating nematodes. Proc. Natl. Acad. Sci. U.S.A. 117, 17913–17923. doi: 10.1073/pnas.1909710117. PMID: 32651273 PMC7395504

[B38] Gomez Gallego S. Loukas A. Slade R. W. Neva F. A. Varatharajalu R. Nutman T. B. . (2005). Identification of an astacin-like metalloproteinase transcript from the infective larvae of *Strongyloides stercoralis*. Parasitol. Int. 54, 123–133. doi: 10.1016/j.parint.2005.02.002. PMID: 15866474

[B39] Grant W. N. Skinner S. J. Newton-Howes J. Grant K. Shuttleworth G. Heath D. D. . (2006). Heritable transgenesis of *Parastrongyloides trichosuri*: a nematode parasite of mammals. Int. J. Parasitol. 36, 475–483. doi: 10.1016/j.ijpara.2005.12.002. PMID: 16500659

[B40] Hallem E. A. Spencer W. C. McWhirter R. D. Zeller G. Henz S. R. Ratsch G. . (2011). Receptor-type guanylate cyclase is required for carbon dioxide sensation by *Caenorhabditis elegans*. Proc. Natl. Acad. Sci. U.S.A. 108, 254–259. doi: 10.1073/pnas.1017354108. PMID: 21173231 PMC3017194

[B41] Howe K. L. Bolt B. J. Shafie M. Kersey P. Berriman M. (2017). WormBase ParaSite - a comprehensive resource for helminth genomics. Mol. Biochem. Parasitol. 215, 2–10. doi: 10.4161/worm.19574. PMID: 27899279 PMC5486357

[B42] Hu P. J. (2007). Dauer. In Riddle D. L. (Ed.), WormBook (Pasadena: WormBook), 1–19. doi: 10.1895/wormbook.1.144.1

[B43] Hunt V. L. Hino A. Yoshida A. Kikuchi T. (2018). Comparative transcriptomics gives insights into the evolution of parasitism in *Strongyloides* nematodes at the genus, subclade and species level. Sci. Rep. 8, 5192. doi: 10.1038/s41598-018-23514-z. PMID: 29581469 PMC5979966

[B44] Hunt V. L. Tsai I. J. Coghlan A. Reid A. J. Holroyd N. Foth B. J. . (2016). The genomic basis of parasitism in the *Strongyloides* clade of nematodes. Nat. Genet. 48, 299–307. doi: 10.1038/ng.3495. PMID: 26829753 PMC4948059

[B45] Hunt V. L. Tsai I. J. Selkirk M. E. Viney M. (2017). The genome of *Strongyloides* spp. gives insights into protein families with a putative role in nematode parasitism. Parasitology 144, 343–358. doi: 10.21956/wellcomeopenres.27461.r132136 27618747

[B46] Junio A. B. Li X. Massey H. C. Nolan T. J. Lamitina S. T. Sundaram M. V. . (2008). *Strongyloides stercoralis*: cell- and tissue-specific transgene expression and co-transformation with vector constructs incorporating a common multifunctional 3’ UTR. Exp. Parasitol. 118, 253–265. doi: 10.1016/j.exppara.2007.08.018 17945217 PMC2259275

[B47] Kim H. M. Hong Y. Chen J. (2022). A decade of CRISPR-Cas genome editing in *C. elegans*. Int. J. Mol. Sci. 23, 15863. doi: 10.3390/ijms232415863 36555505 PMC9781986

[B48] Knott G. J. Doudna J. A. (2018). CRISPR-Cas guides the future of genetic engineering. Science 361, 866–869. doi: 10.1126/science.aat5011. PMID: 30166482 PMC6455913

[B49] Li H. Gazzola D. Hu Y. Aroian R. V. (2023). An efficient method for viable cryopreservation and recovery of hookworms and other gastrointestinal nematodes in the laboratory. Int. J. Parasitol. 53, 451–458. doi: 10.1016/j.ijpara.2023.05.001. PMID: 37201563 PMC10330584

[B50] Li X. Massey H. C. Nolan T. J. Schad G. A. Kraus K. Sundaram M. . (2006). Successful transgenesis of the parasitic nematode *Strongyloides stercoralis* requires endogenous non-coding control elements. Int. J. Parasitol. 36, 671–679. doi: 10.1016/j.ijpara.2005.12.007. PMID: 16500658

[B51] Li X. Shao H. Junio A. Nolan T. J. Massey H. C. Pearce E. J. . (2011). Transgenesis in the parasitic nematode *Strongyloides ratti*. Mol. Biochem. Parasitol. 179, 114–119. doi: 10.1016/j.molbiopara.2011.06.002. PMID: 21723330 PMC3156851

[B52] Lo N. C. Addiss D. G. Buonfrate D. Amor A. Anegagrie M. Bisoffi Z. . (2025). Review of the WHO guideline on preventive chemotherapy for public health control of strongyloidiasis. Lancet Infect. Dis. 25, e146–e152. doi: 10.1016/s1473-3099(24)00595-4. PMID: 39481419 PMC11871984

[B53] Lok J. B. (2012). Nucleic acid transfection and transgenesis in parasitic nematodes. Parasitology 139, 574–588. doi: 10.1017/s0031182011001387. PMID: 21880161 PMC3319118

[B54] Lok J. (2013). piggyBac: a vehicle for integrative DNA transformation of parasitic nematodes. Mob. Genet. Elements 3, e24417. doi: 10.4161/mge.24417 23914309 PMC3681738

[B55] Lok J. B. Shao H. Massey H. C. Li X. (2017). Transgenesis in *Strongyloides* and related parasitic nematodes: historical perspectives, current functional genomic applications and progress towards gene disruption and editing. Parasitology 144, 327–342. doi: 10.1017/s0031182011001387. PMID: 27000743 PMC5364836

[B56] Lopez P. M. Boston R. Ashton F. T. Schad G. A. (2000). The neurons of class ALD mediate thermotaxis in the parasitic nematode, *Strongyloides stercoralis*. Int. J. Parasitol. 30, 1115–1121. doi: 10.1016/s0020-7519(00)00087-4. PMID: 10996330

[B57] Loukas A. Maizels R. M. Hotez P. J. (2021). The yin and yang of human soil-transmitted helminth infections. Int. J. Parasitol. 51, 1243–1253. doi: 10.1016/j.ijpara.2021.11.001. PMID: 34774540 PMC9145206

[B58] Luvira V. Siripoon T. Phiboonbanakit D. Somsri K. Watthanakulpanich D. Dekumyoy P. (2022). *Strongyloides stercoralis*: a neglected but fatal parasite. Trop. Med. Infect. Dis. 7, 310. doi: 10.3390/tropicalmed7100310 36288051 PMC9609954

[B59] Lynn M. K. Morrissey J. A. Conserve D. F. (2021). Soil-transmitted helminths in the USA: a review of five common parasites and future directions for avenues of enhanced epidemiologic inquiry. Curr. Trop. Med. Rep. 8, 32–42. doi: 10.1007/s40475-020-00221-2. PMID: 33552843 PMC7847297

[B60] Magalhães A. R. Codeco C. T. Svenning J. C. Escobar L. E. Van de Vuurst P. Goncalves-Souza T. (2023). Neglected tropical diseases risk correlates with poverty and early ecosystem destruction. Infect. Dis. Poverty 12, 32. doi: 10.1186/s40249-023-01084-1 37038199 PMC10084676

[B61] Malaiwong N. Porta-de-la-Riva M. Krieg M. (2023). FLInt: single shot safe harbor transgene integration via Fluorescent Landmark Interference. G3 13, jkad041. doi: 10.1093/g3journal/jkad041. PMID: 36805659 PMC10151404

[B62] McKenna M. L. McAtee S. Bryan P. E. Jeun R. Ward T. Kraus J. . (2017). Human intestinal parasite burden and poor sanitation in rural Alabama. Am. J. Trop. Med. Hyg. 97, 1623–1628. doi: 10.4269/ajtmh.17-0396. PMID: 29016326 PMC5817782

[B63] McKerrow J. H. Brindley P. Brown M. Gam A. A. Staunton C. Neva F. A. (1990). *Strongyloides stercoralis* - Identification of a protease that facilitates penetration of skin by the infective larvae. Exp. Parasitol. 70, 134–143. doi: 10.1016/0014-4894(90)90094-s 2137091

[B64] Mendez P. Walsh B. Hallem E. A. (2022). Using newly optimized genetic tools to probe *Strongyloides* sensory behaviors. Mol. Biochem. Parasitol. 250, 111491. doi: 10.1016/j.molbiopara.2022.111491. PMID: 35697205 PMC9339661

[B65] Möhrlen F. Hutter H. Zwilling R. (2003). The astacin protein family in *Caenorhabditis elegans*. Eur. J. Biochem. 270, 4909–4920. doi: 10.1046/j.1432-1033.2003.03891.x 14653817

[B66] Monsalve G. C. Yamamoto K. R. Ward J. D. (2019). A new tool for inducible gene expression in *Caenorhabditis elegans*. Genetics 211, 419–430. doi: 10.1534/genetics.118.301705. PMID: 30504365 PMC6366904

[B67] Moser M. S. Hallem E. A. (2024). Astacin metalloproteases in human-parasitic nematodes. Adv. Parasitol. 126, 177–204. doi: 10.1016/bs.apar.2024.03.001. PMID: 39448190 PMC12460076

[B68] Ng’etich A. I. Amoah I. D. Bux F. Kumari S. (2023). Anthelmintic resistance in soil-transmitted helminths: One-Health considerations. Parasitol. Res. 123, 62. doi: 10.1007/s00436-023-08088-8 38114766 PMC10730643

[B69] Nolan T. J. Megyeri Z. Bhopale V. M. SChad G. A. (1993). *Strongyloides stercoralis*: the first rodent model for uncomplicated and hyperinfective strongyloidiasis, the Mongolian gerbil (*Meriones unguiculatus*). J. Infect. Dis. 168, 1479–1484. doi: 10.1093/infdis/168.6.1479 8245532

[B70] Park J. O. Pan J. Mohrlen F. Schupp M. O. Johnsen R. Baillie D. L. . (2010). Characterization of the astacin family of metalloproteases in *C. elegans*. BMC Dev. Biol. 10, 14. doi: 10.1186/1471-213x-10-14. PMID: 20109220 PMC2824743

[B71] Patel R. Bartolo G. Castelletto M. L. Garcia Romero A. Bryant A. S. Agak G. W. . (2025). Dopamine signaling drives skin invasion by human-infective nematodes. Nat. Commun. 16, 7246. doi: 10.1038/s41467-025-62517-z. PMID: 40804046 PMC12350745

[B72] Patel R. Bryant A. S. Castelletto M. L. Walsh B. Akimori D. Hallem E. A. (2024). The generation of stable transgenic lines in the human-infective nematode *Strongyloides stercoralis*. G3 14, jkae122. doi: 10.1093/g3journal/jkae122. PMID: 38839055 PMC11304987

[B73] Riaz M. Aslam N. Zainab R. Ur Rehman A. Rasool G. Irfan Ullan M. . (2020). Prevalence, risk factors, challenges, and the currently available diagnostic tools for the determination of helminths infections in human. Eur. J. Inflamm. 18, 1–15. doi: 10.1177/2058739220959915

[B74] Safer D. Brenes M. Dunipace S. SChad G. (2007). Urocanic acid is a major chemoattractant for the skin-penetrating parasitic nematode *Strongyloides stercoralis*. Proc. Natl. Acad. Sci. U.S.A. 104, 1627–1630. doi: 10.1073/pnas.0610193104. PMID: 17234810 PMC1785286

[B75] Sandoval-Villegas N. Nurieva W. Amberger M. Ivics Z. (2021). Contemporary transposon tools: a review and guide through mechanisms and applications of Sleeping Beauty, piggyBac and Tol2 for Genome Engineering. Int. J. Mol. Sci. 22, 5084. doi: 10.3390/ijms22105084. PMID: 34064900 PMC8151067

[B76] Sengthong C. Yingklang M. Intuyod K. Haonon O. Pinlaor P. Jantawong C. . (2021). Repeated ivermectin treatment induces ivermectin resistance in *Strongyloides ratti* by upregulating the expression of ATP-binding cassette transporter genes. Am. J. Trop. Med. Hyg. 105, 1117–1123. doi: 10.4269/ajtmh.21-0377. PMID: 34339389 PMC8592163

[B77] Shao H. Li X. Lok J. B. (2017). Heritable genetic transformation of *Strongyloides stercoralis* by microinjection of plasmid DNA constructs into the male germline. Int. J. Parasitol. 47, 511–515. doi: 10.1016/j.ijpara.2017.04.003. PMID: 28577882 PMC5538930

[B78] Shao H. Li X. Nolan T. J. Massey H. C. Pearce E. J. (2012). Transposon-mediated chromosomal integration of transgenes in the parasitic nematode *Strongyloides ratti* and establishment of stable transgenic lines. PloS Pathog. 8, e1002871. doi: 10.1371/journal.ppat.1002871. PMID: 22912584 PMC3415448

[B79] Smith E. S. Martinez-Velazquez L. Ringstad N. (2013). A chemoreceptor that detects molecular carbon dioxide. J. Biol. Chem. 288, 37071–37081. doi: 10.1074/jbc.m113.517367. PMID: 24240097 PMC3873563

[B80] Stoltzfus J. D. Bart S. M. Lok J. B. (2014). cGMP and NHR signaling co-regulate expression of insulin-like peptides and developmental activation of infective larvae in *Strongyloides stercoralis*. PloS Pathog. 10, e1004235. doi: 10.1371/journal.ppat.1004235. PMID: 25010340 PMC4092141

[B81] Stoltzfus J. D. Massey H. C. Nolan T. J. Griffith S. D. Lok J. B. (2012). *Strongyloides stercoralis age-1*: a potential regulator of infective larval development in a parasitic nematode. PloS One 7, e38587. doi: 10.1371/journal.pone.0038587 22701676 PMC3368883

[B82] Tamarozzi F. Guevara A. G. Anselmi M. Vicuna Y. Prandi R. Marquez M. . (2023). Accuracy, acceptability, and feasibility of diagnostic tests for the screening of *Strongyloides stercoralis* in the field (ESTRELLA): a cross-sectional study in Ecuador. Lancet Glob. Health 11, e740–e748. doi: 10.1016/s2214-109x(23)00108-0. PMID: 36972722

[B83] Vacca F. Mules T. C. Camberis M. Lavender B. Noble S. L. Cait A. . (2024). Controlled infection with cryopreserved human hookworm induces CTLA-4 expression on Tregs and upregulates tryptophan metabolism. Gut Microbes 16, 2416517. doi: 10.1080/19490976.2024.2416517. PMID: 39411786 PMC11485773

[B84] Viney M. E. Thompson F. J. Crook M. (2005). TGF-β and the evolution of nematode parasitism. Int. J. Parasitol. 35, 1473–1475. doi: 10.1016/j.ijpara.2005.07.006. PMID: 16139836

[B85] Wang H. Liu J. Gharib S. Chai C. M. Schwarz E. M. Pokala N. . (2017). cGAL, a temperature-robust GAL4-UAS system for *Caenorhabditis elegans*. Nat. Methods 14, 145–148. doi: 10.1038/nmeth.4109. PMID: 27992408 PMC5693259

[B86] Yang W. Zhu J. K. Jin W. (2024). A catalog of gene editing sites and genetic variations in editing sites in model organisms. BMC Genomics 25, 1153. doi: 10.1186/s12864-024-11073-9. PMID: 39614172 PMC11606270

[B87] Yoshina S. Suehiro Y. Kage-Nakadai E. Mitani S. (2016). Locus-specific integration of extrachromosomal transgenes in *C. elegans* with the CRISPR/Cas9 system. Biochem. Biophys. Rep. 5, 70–76. doi: 10.1016/j.bbrep.2015.11.017. PMID: 28955808 PMC5600330

[B88] Yusa K. (2015). piggyBac transposon. Microbiol. Spectr. 3, MDNA3-0028-2014. doi: 10.1038/nprot.2013.126. PMID: 26104701

[B89] Yusa K. Zhou L. Li M. A. Bradley A. Craig N. L. (2011). A hyperactive piggyBac transposase for mammalian applications. Proc. Natl. Acad. Sci. U.S.A. 108, 1531–1536. doi: 10.1073/pnas.1008322108. PMID: 21205896 PMC3029773

[B90] Zhang L. Ward J. D. Cheng Z. Dernburg A. F. (2015). The auxin-inducible degradation (AID) system enables versatile conditional protein depletion in *C. elegans*. Development 142, 4374–4384. doi: 10.1242/dev.129635. PMID: 26552885 PMC4689222

[B91] Zhang B. Zhou T. Zhu R. Qin P. Li J. Wang C. . (2025). Aptazyme-mediated gene regulation in *Strongyloides stercoralis* for functional studies of insulin receptor isoform specificity. PloS Pathog. 21, e1013774. doi: 10.1371/journal.ppat.1013774. PMID: 41406115 PMC12711028

[B92] Zhou H. Yuan W. Lei W. Zhou T. Qin P. Zhang B. . (2023). Domain definition and preliminary functional exploration of the endonuclease NOBP-1 in *Strongyloides stercoralis*. Parasit Vectors 16, 399. doi: 10.1186/s13071-023-05940-9. PMID: 37924155 PMC10623843

